# Linking coastal environmental and health observations for human wellbeing

**DOI:** 10.3389/fpubh.2023.1202118

**Published:** 2023-09-14

**Authors:** Paul A. Sandifer

**Affiliations:** Center for Coastal Environmental and Human Health, College of Charleston, Charleston, SC, United States

**Keywords:** coastal health benefits, coastal health threats, health observing system, environmental data, sentinel species, environmental justice

## Abstract

Coastal areas have long been attractive places to live, work, and recreate and remain so even in the face of growing threats from global environmental change. At any moment, a significant portion of the human population is exposed to both positive and negative health effects associated with coastal locations. Some locations may be “hotspots” of concern for human health due to ongoing climatic and other changes, accentuating the need for better understanding of coastal environment-human health linkages. This paper describes how environmental and health data could be combined to create a coastal environmental and human health observing system. While largely based on information from the US and Europe, the concept should be relevant to almost any coastal area. If implemented, a coastal health observing system would connect a variety of human health data and environmental observations for individuals and communities, and where possible cohorts. Health data would be derived from questionnaires and other personal sources, clinical examinations, electronic health records, wearable devices, and syndromic surveillance, plus information on vulnerability and health-relevant community characteristics, and social media observations. Environmental data sources would include weather and climate, beach and coastal conditions, sentinel species, occurrences of harmful organisms and substances, seafood safety advisories, and distribution, proximity, and characteristics of health-promoting green and blue spaces. Where available, information on supporting resources could be added. Establishment of a linked network of coastal health observatories could provide powerful tools for understanding the positive and negative health effects of coastal living, lead to better health protections and enhanced wellbeing, and provide significant benefits to coastal residents, including the historically disadvantaged, as well as the military, hospitals and emergency departments, academic medical, public health, and environmental health programs, and others. Early networks could provide best practices and lessons learned to assist later entries.

## 1. Introduction

Despite growing threats from sea level rise and other accelerating climate and environmental change-associated factors, there has been no noticeable diminution in the numbers of people living in coastal areas, and in fact coastal populations may be increasing (1, 2). Depending on region, roughly one-third to one-half of the global population lives on a relatively narrow ribbon of coastal land adjacent to oceans, seas, estuaries, major rivers, and very large lakes (3), and many more people work and recreate there.

Coastal zones and their urban and rural areas are particularly exposed to and affected by numerous climate change factors as a result of their high population densities (in the US, coastal shoreline and watershed counties have 3–4 times the population density as inland counties), frequent high proportion of older adults, and usually low elevation, and they play significant roles in national economies due to tourism, fishing, ports, and other industries (1, 4, 5). Note that here coastal shoreline counties refers to those that are adjacent to the ocean or another large water body while coastal watershed counties lie immediately behind the shoreline counties. Other coastal areas of the world, particularly in Asia and Africa, have or are expected to have large population segments exposed to climate change-associated risks from coastal flooding (6). Eight of the world’s 10 most populous cities, along with many other important urban areas, are coastal (7), and virtually all of these are at risk from climate change. In Europe alone, over 200 million people live in coastal or river-front cities (8).

Living, working in, or visiting coastal areas can affect one’s health, both positively and negatively. White et al. (9) reviewed numerous studies, most from developed countries, that found associations between better self-reported health and coastal residency and even visits to coastal areas, especially for mental health. Whether these associations hold in more resource-constrained areas is poorly known, particularly in the global South and Asia. However a recent study in Indonesia demonstrated that access to blue space for recreation had positive mental health effects (10). Overall, the weight of evidence for a positive effect of coastal exposure on health is substantial (9, 11–13).

At the same time, health-related exposures that may occur as a result of coastal residence or visits also include a variety of health hazards such as harmful algal blooms (HABs) and their toxins, infectious disease organisms (e.g., naturally-occurring *Vibrio* bacteria and microbes associated with sewage pollution), oil spills, chemicals of known and unknown toxicity in air, water, soil, and seafood, and risks of injury and drowning caused by storms, floods, and rip currents. With sea level rise and extreme weather events, there is increasing evidence of stress-associated psychological and physiological disorders caused by disruption to and/or loss of livelihoods and ways of life, damage to or destruction of housing and treasured places, and disturbance of social and familial networks resulting from coastal disasters (14–18). Some of the health- threatening and health-supporting effects of coastal and ocean environments are the primary foci of Oceans and Human Health (OHH) research programs in the US and EU (19–21).

Millions of people around the world rely on weather forecasts, and to a lesser but growing extent, projections of future climatic conditions. These forecasts are increasingly important as people make decisions about daily life activities, places to live, business, investments, travel, recreation, and protection of life and property. The foundation sources for weather and climate forecasts are environmental observing systems that operate on a continuous basis collecting information on atmospheric, oceanic, weather and climate conditions.

These environmental observing systems, and the computer-based numerical models used to process the resulting data, underpin critical, time-sensitive warnings for tropical cyclones and other significant storms, extreme precipitation events, heatwaves, droughts, other major hazards, and longer-term risks from climate change. In part because of the widespread availability of such warnings, the death toll from weather- and climate-related disasters has decreased markedly over the past several decades, even as the number and severity of such events increased (22).

Unfortunately, similar comprehensive, continuous data collection and analysis systems to monitor human health conditions and enable robust predictions and alerts are not widely available, especially in areas exposed to multiple significant health risks. This lack of baseline health information in the disaster-prone US Gulf of Mexico (GoM) region inspired the development of a framework for a community health observing system (16, 23). The COVID-19 pandemic further exposed the need for more comprehensive health surveillance, particularly in areas with combined human health and environmental vulnerabilities (24–27).

Among recommendations for improving interactions between OHH programs and the public health community, Fleming et al. (28, p. 810) included “*design and support implementation of dedicated OHH indicators, data streams, and repositories*.” Similarly, the European Marine Board (EMB) (29) in the H2020 Seas Oceans and Public Health in Europe (SOPHIE) Project identified a need to determine what available environmental and health data are likely to be most useful in an OHH context. And, among recommendations for US contributions to the UN Decade of the Ocean, the National Academies of Science, Engineering, and Medicine included an “Oceans of Data” theme. This theme envisions not only the collection of much new data but also the open accessibility of existing data and improvements in their usability for diverse audiences (30).

The purpose of the present paper is to elaborate on the potential to link coastal and ocean environmental observations to periodic and ongoing health assessments for people residing in coastal areas, with a view toward improving their health by better identifying and characterizing health benefits and threats to maximize the former and mitigate the latter. The timeliness of this work, at least in the US, is reflected in the recent workshop report from the US National Academies of Science, Engineering, and Medicine (NASEM) about integrating public and ecosystem health programs to foster resilience and the Biden-Harris Administration’s commitment to a “whole-of-government approach to valuing the connections between human and ecosystem health” (31, pp. 2–3).

## 2. Materials and methods

### 2.1. Background

**Figure 1:**
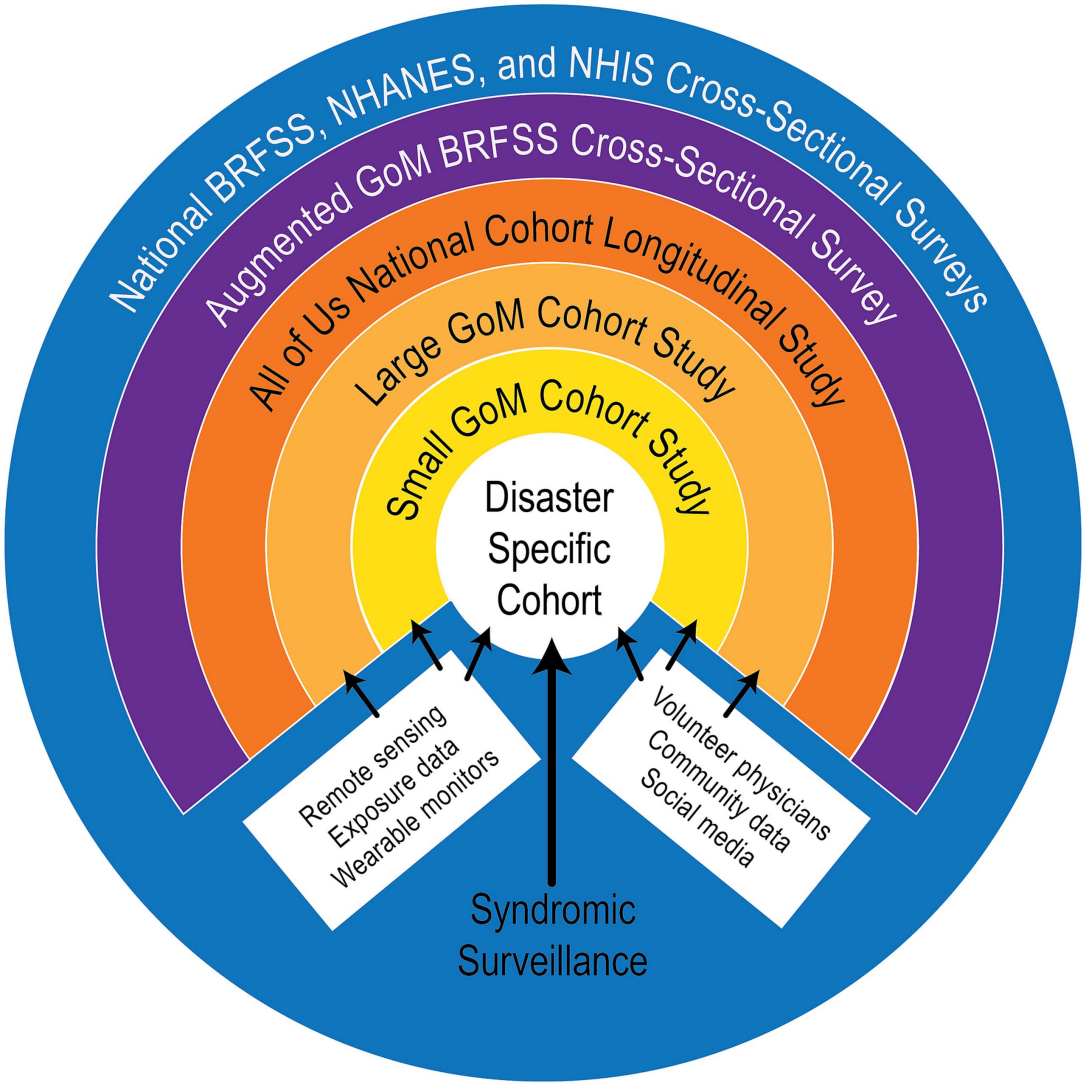
Diagram of a conceptual framework for a Gulf of Mexico Community Health Observing System [from Sandifer et al. (16) open access paper, used with permission of the first author].

A previously published GoM Community Health Observing System framework (16) for disaster-related health monitoring was used as the starting point for conceptualization of a coastal environmental and health observing system (Figure 1). That framework incorporates national, mostly cross-sectional, human health and community surveys [e.g., the National Health and Nutrition Examination Survey], the Behavioral Risk Factor Surveillance System (BRFSS), and the National Health Interview Survey (NHIS) (outer blue ring), a proposed augmented BRFSS study including additional questions relating to disaster exposure (purple ring), plus the National Institute of Health’s (NIH) All of Us longitudinal study (orange ring), and other resources. These data would provide background information about population health and community characteristics for comparison with information derived from the three proposed longitudinal cohort studies (gold, yellow and white circles). The proposed cohort studies are the heart and most critical part of the planned GoM human health observing system (Figure 1).

As proposed, the GoM cohorts would consist of volunteer participants organized into large, small, and disaster-specific cohorts. Personally-provided and clinically-derived health information for cohort participants would be augmented with available observations from a variety of additional sources including remote sensing, wearable health monitors, social media, and others (Figure 1).

The large cohort is intended to be representative of the entire population of a region (e.g., the US GoM coastal counties or in the present context any coastal region or sub-region). Participants in the large cohort would be expected to complete detailed questionnaires about their personal and family health, including demographic and socio-economic information, as well undergo clinical evaluations for physical and mental health.

Members of the small cohort would be solicited from the large cohort, and its members would be expected to provide more clinical data. These volunteer participants would allow collection of biological specimens (e.g., blood, urine, nasal swabs, and saliva samples) that could be analyzed for biomarkers of health conditions and provide access to their electronic health record (EHRs).

Finally, a disaster-specific cohort would be recruited based on the primary affected area of a disaster such as a major storm or flood. Such a cohort would be established as rapidly as possible following a given disaster. It would include any members of the large and small cohorts affected by the disaster, first responders and disaster workers, as well as new volunteer recruits from the disaster area who would agree to provide detailed health information and clinical evaluations.

In non-disaster contexts such as envisioned here, group-specific cohorts could be focused on small geographic areas (e.g., a specific coastal community or area) or common demographic characteristics (e.g., age, gender, socio-economic status, ethnicity). These could also include people who suffer certain chronic conditions (e.g., diabetes, heart disease, obesity), refugees, or those who have experienced major disasters, pandemics, environmental injustice, or the trauma of war or other systemic violence (23). Members of all cohorts would be expected to continue participation, with periodic health assessments via questionnaires and clinical visits, over a long period of time.

Data from the national surveys and cohort studies would be supplemented with information provided from other means as noted in the bottom quadrant of Figure 1. Several of these are explained in more detail in the results section. Note that the category “volunteer physicians” should be interpreted more generally as “volunteer health care providers” including Nurse Practitioners, Physician Assistants, and others. With regard to the following proposal for a coastal environmental and health observing system, when the system approaches an operational state, health care volunteers would be enlisted from local practitioners including health departments, hospitals, and private practices and cohort participants would be recruited from communities (see 16 for suggested cohort recruitment and retention protocols).

### 2.2. Environmental data

Next, the kinds of coastal environmental data that are available now or may become available in the near future and their relevance in health contexts, as well as significant data gaps, were considered. One source of such information is a recent book, *Preparing a Workforce for the New Blue Economy* (32). Although more focused on data streams that could be of economic value, many also could be useful in health assessments, including information from oceanographic, ecological, biodiversity, eDNA (environmental DNA), and other studies.

Other sources of ocean and coastal environmental information are summarized in (33), and an example of linked coastal observations in the environmental arena is provided by the US Integrated Ocean Observing System (IOOS) (34). IOOS^®^ is a system of 11 connected, regional ocean and coastal observing programs that encompass most of the US coastline and provide significant additional information for weather and climate models and predictions. Numerous other sources of environmental information are included in the following Results section.

## 3. Results

**Figure 2:**
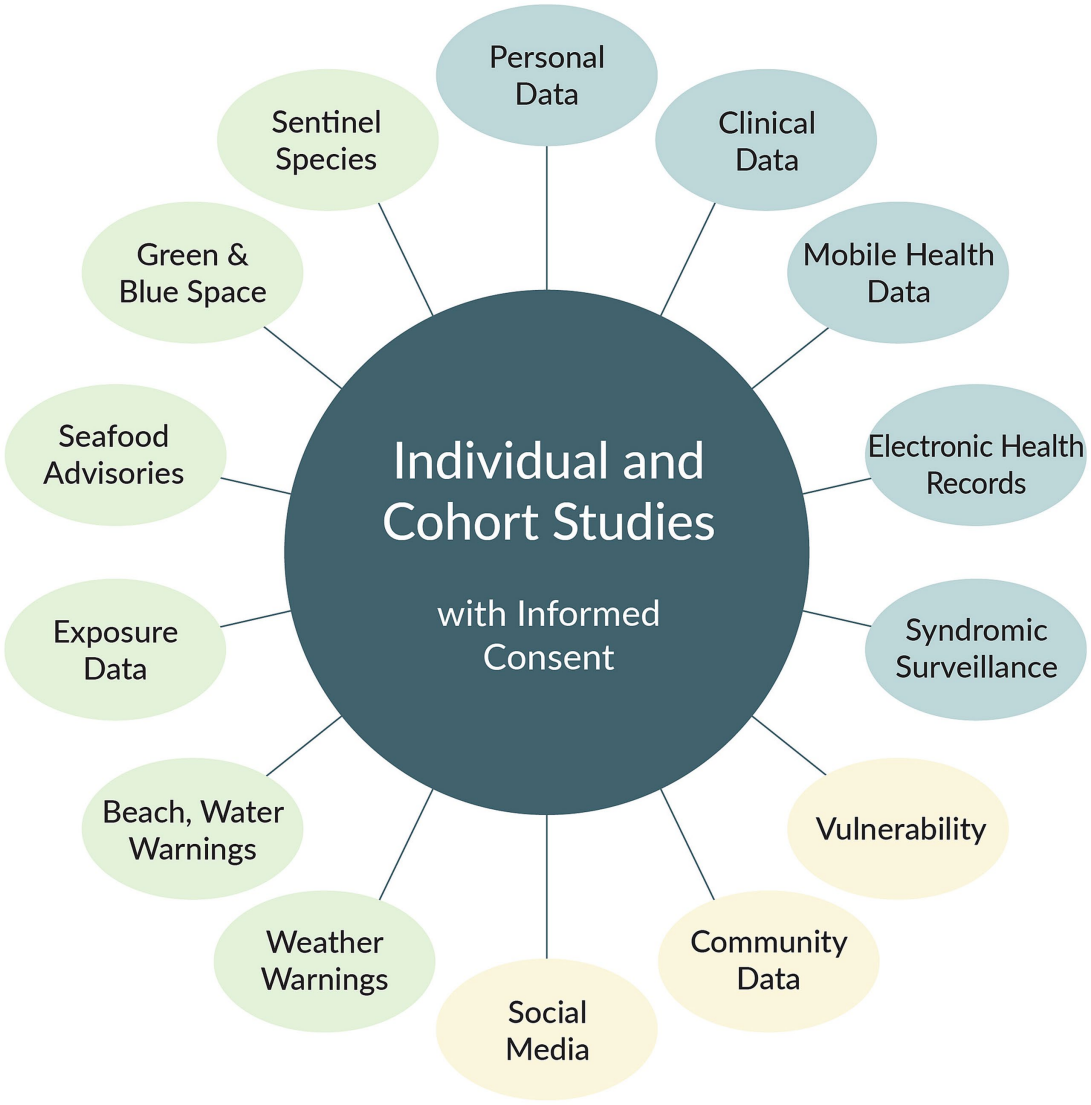
Schematic diagram of a possible future Coastal Environmental and Human Health Observing System. Blue color refers to health data derived from specific individuals; light tan denotes data for individual people and communities; light green denotes ancillary data from warnings, forecasts, and environmental characteristics. (copyright P.A. Sandifer, used with permission). The color scheme used here is not related to that used in Figure 1.

A schematic diagram of components of a hypothetical Coastal Environmental and Human Health Observing System is provided in Figure 2. While certainly not all inclusive, this is an exemplar of the kinds of environmental and health information that could be accumulated and linked for human health assessments. In addition to the items listed, information on supporting resources, e.g., health care facilities and services, community food pantries, emergency response, and others also could be included, where possible using interactive geospatial mapping frameworks [for example see (35)]. The proposed elements would go a long way to providing a more comprehensive view of coastal exposures likely to have positive or negative health consequences. The types of information encompassed by each “bubble” are described briefly following Figure 2.

Similar to the proposed GoM health observing system framework, background and comparative data could be provided by national health surveys already described for the US (16) or similar surveys in other countries (e.g., (36–39) and numerous others). These types of studies provide regional-to-national level data on general health characteristics of populations that can be used for comparison with results from more focused studies. While useful for comparative purposes in some contexts, these data sources have a variety of weaknesses, including issues related to their geographic and temporal scales.

Cross-sectional studies generally do not assess the health of the same individuals over time but use statistically based sampling methods to choose different participants each time the study or survey is conducted (e.g., annually). Unfortunately, while cross-sectional studies can identify associations between factors and observed health characteristics, they are not able to determine cause-and-effect relationships. Identification of causation requires knowledge that an exposure or behavior preceded the health outcome, while accounting for other potential driving variables. Modern molecular and statistical tools can improve epidemiologists’ ability to use certain cross-sectional data to infer causation (40), but long-running longitudinal cohort studies are the “gold standard” for linking cause and effect. However, cohort studies are expensive and difficult to establish and maintain. Although cohort studies would be preferred elements of the observing system, even if cohorts are absent, all the information gathered could be used by individuals, families, and communities for their own health-monitoring. Here, individuals and cohorts are shown in the center of the framework schematic, and each of the elements in the surrounding “bubbles” is elaborated by reference number in the following sections.

A cohort (#1 in Figure 2) is a group of people each of whom agree to participate in periodic health assessments over a relatively long period of time or indefinitely. Depending on the objectives for a given cohort study, individual participants may be chosen to represent a defined population or specific characteristics such as gender, ethnicity, behaviors (e.g., smokers vs. non-smokers), or health status.

For the US, the Health Insurance Portability and Accountability Act of 1996 (HIPAA) spells out patients’ rights to privacy and to control access to and use of their health data (41). Based on HIPAA, the concept of “informed consent” is an essential requirement for cohort and individual health studies. It refers to communication between a physician, other health-care professional or researcher and a patient or study participant which results in clear understanding by the patient/participant of what a specific intervention or study would entail. It culminates in written authorization by the patient/participant for their participation and the use of any data and/or samples derived from the individual (42). The informed consent process defines what data and/or biological specimens would be collected, managed, protected, and used. However, while cohort studies are the preferred basis for long-term health monitoring, researchers and health care personnel may experience considerable difficulties in recruiting and retaining sufficient volunteers (43, 44). These and other difficulties must be considered when planning recruitment efforts. One frequently used method to engage with communities and solicit volunteer input to identify health indicators, data sources, and other information is through carefully designed and facilitated workshops and community meetings (16, 45, 46). It is likely that this approach also could be used to identify willing participants. In the absence of cohorts, data can be accumulated for individuals, families, or small communities over whatever time period is possible. The key will be to enroll sufficient numbers of volunteer participants, whether acting as individuals or as members of a cohort, who will continue to provide health data over a period of time to develop an ongoing baseline against which future health data can be compared.

Personally provided information (PPI) (#2) is derived from detailed questionnaires completed by volunteer participants online or digitally on computers, tablets, or phones, or on paper (not preferred unless the paper records can quickly be digitized), and with or without a trained interviewer to assist with any questions. Desired information includes: demographic and socio-economic information, personal health status, personal and family health and trauma history, behavioral factors such as smoking and sleep habits, health care access, medications, housing status, known exposures to toxic or infectious agents, adverse childhood experiences, social connections, marginalization and/or discrimination, and feeling secure or insecure in one’s home and neighborhood. Similar kinds of health questionnaires are utilized in many national health surveys. Completed digital PPI questionnaires provide a wealth of health-related background information that can be stored, managed, shared and analyzed readily (16). For example, the US Centers for Disease Control and Prevention (CDC) has managed such data from the BRFSS and NHANES surverys for decades, and the data have been used in many studies by academic as well as government scientists.

Mental and physical health assessments (#3) are typically carried out in-clinic using a variety of psychological evaluation instruments and measurements of physical factors (e.g., blood pressure, pulse rate, height, weight, body mass index) and information derived from biological samples (e.g., blood, urine, nasal swabs, and saliva) (16). However, in certain situations such as emergencies, pandemics, or in rural areas where clinic visits may be difficult, telemedicine or telehealth approaches can be used for some assessments and the completion of questionnaires. Both telemedicine and telehealth refer to the provision of medical services via electronic means, typically video conferencing between patients and health care providers. Its use and acceptance by health professionals and the public have grown substantially over the last several years, especially during the COVID-19 pandemic (47–50).

**Figure 3:**
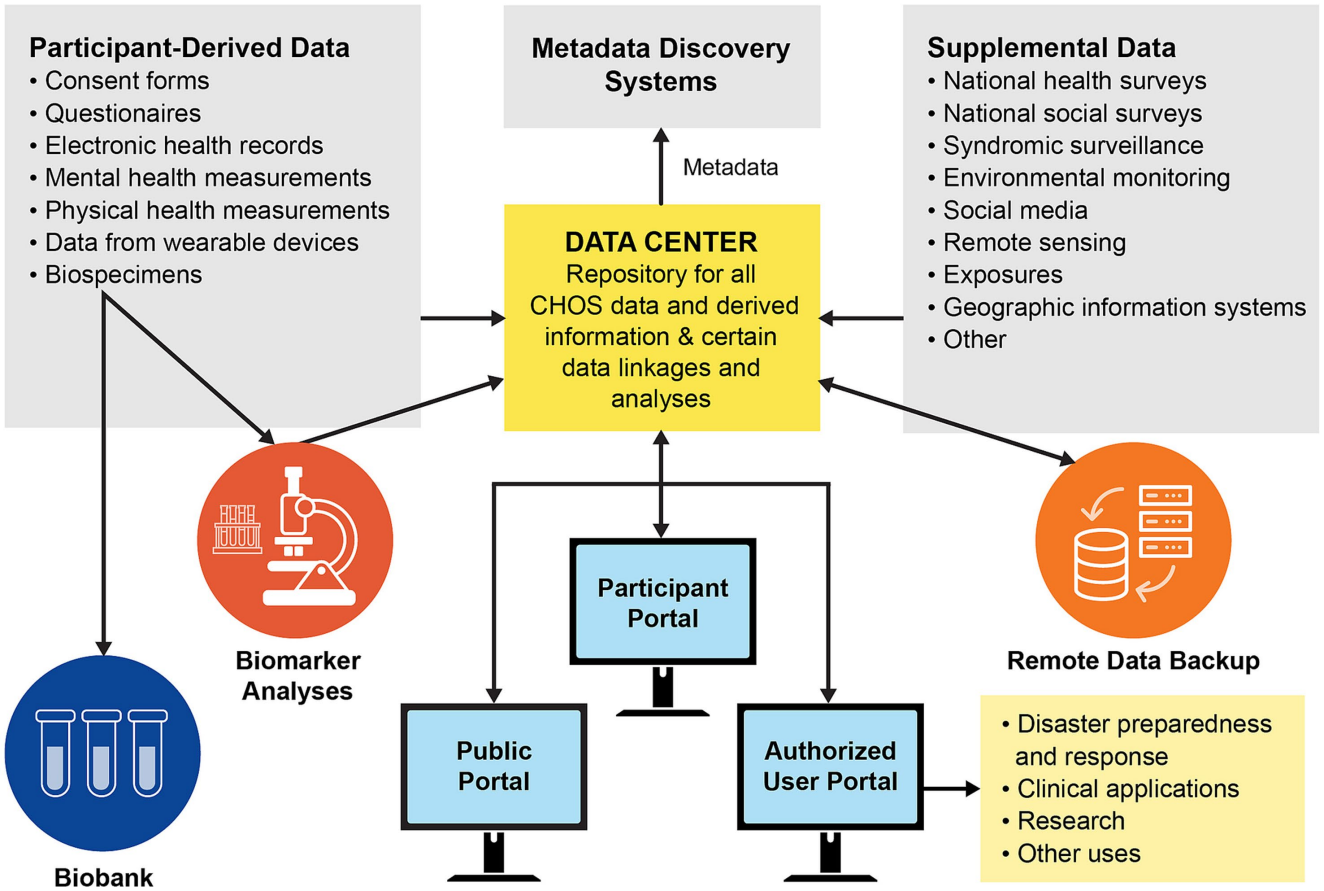
Data and specimen management system for a future GoM Community Health Observing System (biobank refers to long-term frozen storage of biological samples for later analysis) [from Sandifer et al. (16); open access paper, used with permission of the first author]. A similar approach could be followed for the Coastal Environmental and Health Observing System envisioned here.

In-clinic health assessments may also incorporate measurements of cumulative and harmful psychosocial and physiological stress (i.e., allostatic load) via specially designed psychological questionnaires and physiological biomarkers derived from biological specimens (51). Biological specimens collected would be deposited in a secure biobank for use, with all necessary and appropriate safeguards and standardization of methods (Figure 3).

Mobile health data (#4) are those that can be collected via use of personal smartphone apps and/or wearable health devices that can monitor, record, and in some cases transmit data for parameters such as heart rate and heart rate variability, activity (e.g., walking), sleep, blood pressure, respiration, glucose levels, and others (16). A variety of wearable health devices are already available with many improvements and new devices in the pipeline (52). Mobile devices selected for use in this context should be readily accepted, affordable, willingly used by people, robust in a wide range of environmental conditions, and have dependable and long-lasting power sources.

Mobile phones are the most ubiquitous of mobile devices, and they would likely be the primary target for a coastal health observing system. In the US, ~96% of adults have a cell phone (53). At the global level, ~92% of people have a mobile phone, and 84% of these (6.64 billion) have a smartphone (54). Estimates of the number of health-related apps for smartphones range from ~85,000 to 259,000 (55, 56), with more being developed.

Pairing of smartphones and smartwatches or other devices can make possible continuous monitoring of certain health parameters, along with data on location and the physical environment (temperature, humidity, wind, etc.). Mobile devices also can be adapted to include periodic responses regarding psychological status (57) while experiencing a coastal environment for example or for short self-report surveys, such as reactions in the presence of a HAB event, oil spill, hurricane, etc. (43, 58, 59).

Electronic health records (EHRs) (#5) are digital files maintained for individual patients by health care providers and hospitals and subject to strong privacy protections. An EHR is a continuously running summary of an individual’s health information, as determined via in-person and telehealth visits, prescriptions, questionnaires, etc. and is considered a basic repository of patient data. It is effectively a longitudinal record of an individual’s health information. Data derived for individuals participating in cohort studies can be linked to their EHRs with informed consent of the participant. These data can then be integrated with all the individual’s other health information to allow health-care professionals and/or the individual to develop a more comprehensive assessment of one’s health status and health risks. For example, if HAB exposures and associated illness could be better documented in EHRs, they could be linked to other health data over an individual’s life course and thus allow for monitoring of long long-term health effects of such exposures, which are poorly known at present (60). This could be particularly important for children and other vulnerable populations. Some organizations have developed EHR sharing programs, with consent of the participating individuals (61, 62). Development of a secure sharing platform for EHRs would be an important step for cohort studies and for communities interested in undertaking their own community-wide health monitoring effort (Figure 3).

Syndromic Surveillance (SyS) (#6) is a public health early warning system in US states where digital health information about certain diseases can be gathered rapidly, usually from hospital or other emergency departments, and used for early detection of disease outbreaks. These are commonly used to identify and track outbreaks of infectious diseases such as influenza (63) as well as other hazards (64, 65). However, they are lacking in some important respects, including reporting of mental health issues (16).

From a coastal perspective, SyS has been used to identify Ciguatera poisoning outbreaks caused by harmful algae in Florida (66) and for assessing other HAB-associated illnesses across the US (60). Lavery et al. (60) highlighted the potential value of SyS and EHRs for improved documentation of HAB illnesses. Similarly, more robust SyS linked to EHRs could be used to identify *Vibrio*- and pollution-associated illnesses or other kinds of exposures in coastal areas.

Vulnerability (#7) refers to factors that increase susceptibility of individuals and communities to adverse effects from stressors and exposures (67–69). Basically, vulnerable population segments, groups, or individuals are those who have a higher risk of or susceptibility to injury, illness, death, or loss than the average for the population as whole. Such higher risk may arise from demographic and related characteristics such as age (the very young and the older adult/adults), gender (female), pregnancy, minority race or ethnicity, lower socio-economic and/or educational status, poverty, deprivation, being a refugee, sexual identity and orientation, physical or mental impairment, presence of chronic disease, being institutionalized, incarcerated, or marginalized by language or other factors. Vulnerability also may be defined in terms of proximity to a hazard (e.g., being in the path of a hurricane, tornado, or tsunami or living near highly polluted areas, dangerous industrial sites, or major transportation arteries with their associated pollution, or living or recreating near a large HAB). Here, vulnerability includes those who are at risk due to personal and socio-economic characteristics and/or hazard proximity.

Resiliency is defined in numerous ways, so much so that eventually dissimilar fields may even describe it differently (70). It is probably most often used in reference to the ability of a person or community to resist/absorb stressor impacts and recover rapidly (14). In an attempt to reconcile terms such as adaptability, sustainability, resilience and others in relation to systems facing threats, Galaitsi et al. (71, p. 7) offered the following similar definition: resiliency is the “capacity to recover critical functions and adapt following a disruptive event.” And in the context of social-ecological systems, Reyers et al. (72, p. 269) considered resilience as “the ability of people, communities, societies, or cultures to live and develop with changes and with ever-changing environments. It is about cultivating the capacity to continue to develop in the face of change, incremental and abrupt, expected and surprising.” Relative vulnerability and resiliency can be assessed for both individuals and communities and the information included in EHRs and other health records.

As used here, community data (#8) refers to detailed information about a given community or group of communities, including socio-economic and demographic characteristics, population conditions and health status, resilience, availability and condition of housing, and numerous others. Examples of robust sources of community data in the US are the ACS (73), the General Social Survey (74), and county health data (75). Summers et al. (76) developed a human wellbeing index for the US based on social, economic, and environmental data from secondary sources. Like vulnerability information, these data provide context and help identity factors that may predispose a given individual or group to certain health conditions, positive or negative. Relative indices of resilience to acute weather events at county and regional levels are also available (77, 78). In addition to these secondary sources, community data also encompass local, traditional, and indigenous knowledge brought forth by community members about their environment, health, and interconnections between them. The observing system should include mechanisms for collection of such information and its integration with geospatial and other data derived via scientific approaches (79–82).

Social media (#9) typically refers to websites and applications that enable users to create and share content or participate in social networking (Oxford Language). Common examples include Facebook, Instagram, Twitter, TikTok, WhatsApp, and YouTube. Social media can be used to assess mental and physical health and pollution issues in communities or other geographic areas at specific times, depending on the extent to which the social media data reflects characteristics of a given community (83–85), and to distribute helpful information. However, social media also can be used to marginalize or demonize certain ethnic groups (86) and to spread harmful disinformation, such as occurred during the COVID-19 pandemic (23). In addition, social media can help people establish and maintain social and family networks and friendships, which can exert positive influences on health. For example, people with higher levels of social contact and social capital tend to feel healthier, mentally and physically (87), although social capital may not be protective of physical health in all cases (88). Because social media can be both helpful and harmful, care must be taken in its use for observing and reporting health information. Importantly, social media users may not include proportionate representation of particularly vulnerable populations, such as older adults.

Weather warnings (#10) are used here as an abbreviation to encompass atmospheric, meteorological, oceanographic, and climate observations and warnings. Common examples include weather forecasts, extreme heat, cold and humidity alerts and warnings, and forecasts of tropical cyclones and other major storms, floods, tornados, tsunamis, droughts, and high wave and rip current warnings.

Weather forecasts and other weather and climate-related services are provided by a variety of organizations, including at international [e.g., (89–91)] and national levels [e.g., (92, 93)], and by a plethora of private sector companies, with some tailored to very specific audiences (e.g., farmers, beachgoers, surfers). In the US, Americans consulted weather forecasts approximately 300 billion times per year, illustrating their high value to the public (94).

Beach and water warnings (#11) are advisories and forecasts such as for the presence of HABs and their toxins, sewage pollution, other contaminants, and high concentrations of *Vibrio* bacteria in recreational waters and drinking water supplies. Multiple modeling tools are available for forecasting HABs and *Vibrios* and the accuracy and coverage of these systems are increasing rapidly (95–98). In addition, the US CDC supports the One Health Harmful Algal Bloom System (OHHABS), a voluntary reporting system for states and territories to contribute information on incidences of HABs and associated illnesses in humans and animals (99, 100). While this system typically does not provide data in real or near-real time, it does enable discovery of major HAB events and associated health impacts in a variety of states. It is worth noting, however, that the majority of HAB reports in the system come from freshwater.

The “How’s the Beach” app (101) is one example of internet-based tools that provide beachgoers with information to judge whether a particular day might be a good day to go to a specific beach in terms of conditions that might affect health. This product pulls together weather information (temperature, UV conditions), beach conditions (tides, surf, waves, rip currents), likelihood of exposure to *E. coli* or other pollution-associated disease organisms or HABs, and other relevant data and makes it available in near-real time to consumers via their mobile phones, tablets, or computers.

Exposure (#12) is a catch-all for detection of potential contacts with noxious, toxic, and infectious agents in air (e.g., aerosolized HAB toxins, fine particulate matter) and water (infectious disease organisms, chemical pollution, oil spills, pesticides, herbicides) using direct sampling and remote sensing techniques. Concentrations of the agent(s) of concern and duration of exposure are of particular importance as is the cumulative total exposures (termed the “exposome”) over a lifetime (102).

In addition to exposure data collected locally in coastal areas by environmental agencies, researchers, and citizan scientists, there are numerous other sources of data on water- and airborne exposures [e.g., (103–106)]. In the US, the National Institute of Environmental Health Sciences (NIEHS) maintains the Human Health Exposure Analysis Resource (107) that provides detailed resources for researchers to use in assessing pollution exposure impacts on health. Other important exposure resources are poison control centers located across the US and in other countries (108).

Seafood advisories (#13) are assessments of the likely healthful or harmful status of specific seafood in terms of nutritional quality (e.g., promoting cardiovascular health and safe pregnancies) and/or contaminant burden (heavy metals, HAB toxins and chemical pollutants, *Vibrios* and other infectious microbes) that carry increased risks for a variety of adverse health outcomes, including cancer and birth defects. These notices are often promulgated by health agencies with notices in public media and may cover species harvested in recreational, subsistence, and commercial fisheries and distributed via informal networks or commercial markets (109, 110).

Exposure to “green,” “blue,” and biodiverse landscapes (#14) is widely recognized to have health-promoting effects, especially for mental health (e.g., alleviation of anxiety and depression). Green spaces include forests, parks, tree-lined streets, and other areas relatively rich in trees and natural biota in urban as well as rural areas. “Blue” spaces refer to large water features, especially coastal areas but also large lakes and rivers. While mechanisms of action for health-promoting effects of green and blue spaces are largely unknown, recent studies point to positive effects on immune system function (111), improved mental health and decreased exposure to pollution (112), and increased physical activity and restoration (referring to “recovery from depleted attentional capacity or stress”) (9, 113). In addition, while adverse childhood experiences are known to negatively affect health later in life (114), blue space exposure in childhood has been shown to positively influence adult subjective wellbeing (115). Reduction in COVID-related mortality and racial disparities in COVID infection rates also were reported in association with green spaces (116–118).

A variety of remote sensing data can be used to assess greenness, including the Normalized Difference Vegetative Index (NDVI), Leaf Area Index (LAI), and Land Use- Land Cover (LULC) data. Labib et al. (119) used all these to develop a composite Greenspace Availability Exposure Index (GAVI). Amounts and proximity of blue spaces can also be determined with aerial and satellite imagery. Zaldo-Aubanell et al. (120) make a strong case for linking environmental information such as LULC data with health information maintained in individual EHRs.

Monitoring of sentinel organisms (#15) such as filter-feeding mollusks that often capture pathogens, chemical contaminants, and HAB toxins and marine mammals that breathe the air just above the surface of the water and feed on the same or similar species that humans consume, can provide excellent indicators of the condition of marine and coastal environments and of potential health risks for humans (121, 122). Created in 1986, NOAA’s Mussel Watch program is the longest continuously running program using molluscan shellfish to monitor chemical contaminants in US and Great Lakes coastal waters (123). The program spread internationally (124) and has provided a rich and consistent database on chemical pollution in coastal waters in numerous countries for nearly five decades. Similarly, marine mammal health studies provide valuable insight into effects of chemical contaminants including oil and HAB toxins on organs and life processes as well as on distributions of pathogenic organisms in response to climate change. Observed health effects may indicate how similar exposures could affect humans (125). As one example, reports of illnesses and deaths among livestock, dogs, and fish exposed to HABs can provide useful information about human health risks if shared with public health and clinical practitioners (126). Such reports can be found in the OHHABS system previously mentioned and should be included in health and environmental surveillance systems (126).

Missing from the coastal health observing system schematic (Figure 2) is a means for assimilating, linking, integrating, and maintaining data and associated metadata from all the information streams. While a secure data management system will be an essential element of a coastal health observing system, design of a data warehouse and management system is beyond the scope of the present paper. However, one option would be to establish a secure data repository similar to that employed in the NIH All of Us national health study (127) and proposed for the GoM Community Health Observing System (Figure 3) (16). Such a third-party repository could provide data quality control, storage and archiving, integration, and controlled access. The repository would limit access to protected data only to participants and pre-qualified researchers and health care professionals under strict use protocols. Individual participants could access their specific health data as well as the publicly available environmental data to gain a better understanding of their various exposures and potential associated positive or adverse health effects.

The data repository should also include a “biobank,” that is, protected frozen storage for biological specimens collected from volunteer participants. Utilization of specimens for analysis for biomarkers and other purposes would be limited to authorized persons only, and all privacy requirements for use of the biological materials and all data derived from them would be followed. Some other models for secure systems for storing and managing health information and its use include the UK Biobank (128), My Clinical Outcomes (129), the Opal Project (130), the University College of London’s Data Safe Haven (131), and Data Shield (132).

Because the Coastal Environmental and Health Observing System is just a proposal at this time and lacks a confirmed home and implementation strategy for start-up and operations, governance and decision-making responsibilities can be described only in general terms. Detailed design and implementation of the system will likely require commitment of at least one lead sponsoring agency or entity, perhaps accompanied by establishment of a consortium of several institutional partners to serve as principal funders and as an oversight or managing body. However structured, the managing body would need to take responsibility for final system design and implementation, financial administration, and data management including establishment of data and metadata standards, data sharing agreements, data access and use, as illustrated in Figure 3. It would also be responsible for creating and sustaining scientific and community advisory committees and arranging for necessary periodic reviews and audits.

## 4. Discussion

Coastal areas remain attractive places to live, work, and recreate, even in the face of growing threats from climate and other environmental change. Because so many people are attracted to coasts worldwide, at any given time a significant portion of the human population is exposed to positive and negative health effects associated with coastal location.

More older people are moving to coastal locations. Climate Central (133) reported that the numbers of people >65 years old living in US coastal areas grew by 89% between 1970 and 2010, reflecting at least in part the attraction of coastal areas for retirees. Further, the EPA (134) estimated that, in the US, those 65 and older had a 9% higher chance of living in areas of potential high impact from coastal flooding. This trend of a “graying” coastline is not limited to the US, but also reported in the UK (135) and Mediterranean Europe (136), and it is probably occurring elsewhere. Older people may be able to benefit from coastal health-promoting factors while also likely being more vulnerable to coastal health risks.

Reported health benefits of coastal living are beginning to be explored systematically (137, 138), although mechanisms of action remain poorly understood. Similarly, while many health risks that may accompany coastal residence are well known, their contributions to morbidity and mortality are not, nor are most people even aware of their exposures to potential health threats when at or near the coast.

As examples, human illnesses caused by HABs and infectious *Vibrios*, either via consumption of contaminated food or contact with water or aerosols containing toxins or microbes, have been recognized for decades (139, 140). Such negative effects appear to be increasing in response to climate change-associated rising water temperatures, salinity changes in coastal areas, and interactions of microbes and HABs with nutrients, heavy metals, antibiotics, and microplastics (1, 20, 141). Yet these adverse health outcomes remain under-reported, frequently misdiagnosed, and poorly recognized by medical and public health professionals, as well as by coastal managers, residents, and visitors. For instance, HAB-related illnesses are believed to be at least 10-fold more prevalent than reported (142), while ciguatera poisonings in Florida are estimated to be 55–87 times higher (143). *Vibrio*-associated illnesses are also severely underreported, and in one instance *V. vulnificus* infections were estimated to be 142 times higher than reported (20).

Raising awareness among medical professionals and the public will help in this regard as will improvements in diagnoses and reporting systems and in ecological forecasts that provide early warnings of likely problems (19, 28, 139). Among the most important needs are collection and wide dissemination of information about the occurrence of specific risk factors (e.g., microbial and chemical pollution, HABs, *Vibrios*) and potential human exposures to them. A coastal health observing framework such as proposed here could help fill this gap by bringing attention to environmental and other conditions that can significantly affect people’s health and wellbeing.

### 4.1. Examples of potential users

Central questions are who or what could utilize a health-relevant, coastal data gathering system and how would they do so? Although no specific targets for initial implementation have been identified, there are numerous potential users. A few candidates are introduced briefly below.

Worldwide the US Department of Defense has >1,700 coastal facilities (144), and many active-duty personnel and their families, civilian employees, retirees, and others are routinely exposed to a wide range of coastal environments and conditions. The US military also has a massive medical system (145) and maintains long-term health records for its personnel and dependents. In addition, the US Veterans Health Administration (146) operates 171 medical centers and over 1,000 outpatient care sites, and many of these facilities are located in coastal areas.

The debilitating effects of exposures to HAB toxins, infectious microbes, and chemical contaminants, along with the known salutary employment of nature-based therapies, including blue spaces, to assist in recovery from trauma (PTSD) and injury (147–150), should make a coastal health observing system attractive to the military. In addition, the value of the military’s long-term health data in relation to an environmental disaster was demonstrated in follow up studies regarding impacts of the Deepwater Horizon oil spill in the Gulf of Mexico to US Coast Guard members who worked the spill (151). Adding coastal environmental information to its continuous collection of health data might assist the military in improving diagnostic capacity and treatments.

Private and public civilian hospitals, health care centers, and academic institutions with medical, public health, and environmental health programs are also located in many coastal cities around the world. Any of these could provide opportunities for implementation and/or integration of coastal environmental data with their long-term patient health data.

In the US, the OneFlorida+ Clinical Research Network (62) provides an example of a large consortium of health care facilities, academic institutions, and practitioners, and includes strong participation by “citizen scientists,” that might profit from inclusion of all or at least some elements of a coastal health observing system. This organization covers the state of Florida, most of which is coastal, along with some areas in Alabama and Georgia.

In the UK, a group of medical and environmental experts has formed Healthcare Ocean Ltd. (152) with a vision to “*conserve and protect coastal and marine ecosystems through minimising harm resulting from the procurement and delivery of healthcare whilst increasing awareness of the benefits to human health and wellbeing from healthy seas, coasts, and waterways*.” This kind of organization might also benefit by connecting with a variety of coastal environmental data streams.

Implementation of the coastal health observing system concept, in part or whole, at the level of coastal fishing and tourism communities, neighborhoods, and disadvantaged, marginalized, and environmental justice communities could provide opportunities to establish ongoing, community-wide assessments of factors that might affect the health and wellbeing of their residents. For example, the proposed observing system could build upon and add much information to ongoing European efforts to address linkages between environmental conditions and human health in coastal communities (8, 153, 154). Further, using community-based participatory or community-engaged research approaches (CBPR or CEnR) (155–157), members of a particular community could work with one or more community-based organizations (CBOs) and research partners to implement their own health observing system based on monitoring of a selected suite of environmental exposure parameters and collection of health and community data. Approaches involving application of CPBR with inclusion of CBOs have proved effective in dealing with COVID-19, flooding, and climate change concerns (35, 158, 159). In addition, involvement of the public through citizen science efforts related to health can be highly effective (160).

A specific example of a coastal locality where the coastal health observing system concept might be useful is the Puget Sound area of Washington state in the US. The Puget Sound Partnership is a well-established program focused on monitoring, maintaining, and improving the health of the Sound and its inhabitants and communities. Its principal funder is EPA’s National Estuary Program, but it also includes numerous other contributing partners. The program monitors a wide range of indicators termed “vital signs” including water quality, habitat status, and human wellbeing with an emphasis on environmental justice and equity (82). The coastal health observing system framework might provide opportunities for augmentation of existing monitoring and related efforts.

Impacts of pollution and toxic wastes, plastics and marine debris, climate change, and degradation of ecosystems, biodiversity, and ecosystem services interact, producing cumulative and synergistic effects on the health and wellbeing of coastal communities and people, especially those already socio-economically disadvantaged, marginalized, and isolated (161). While not a solution by itself, the health observing system described here could help alleviate some of the adverse health outcomes and catalyze actions to reduce coastal health hazard risks and impacts, including to the disadvantaged.

A core need identified early in the development of OHH programs and continuing today is for interdisciplinary and trans-sector research and action involving not only marine scientists but also researchers and practitioners in public health and medicine (28, 139, 162). However, gaining the attention of medical and public health professionals and attracting them to OHH has proved to be incredibly challenging, in part likely due to limited funding. Despite efforts in both the US and Europe to do so, this challenge remains largely unanswered (162) and much remains to be done (163, 164). The coastal health observing system could play an important role in accelerating the integration of OHH programs with public health and clinical practice by increasing the reporting and visibility of coastal ocean-related illnesses and health benefits and providing continuous and reliable sources of information on ocean health-associated effects ready at hand.

Finally, while a coastal health observing system is only aspirational now, the “How’s the Beach” web-based tool (101) is an example of early steps in the direction of such a system. It is noteworthy that the tool was developed by a team from the University of South Carolina’s Arnold School of Public Health, the University of Maryland’s Center for Environmental Science, and the Southeast Coastal Ocean Observing Regional Association (SECOORA). SECOORA is one of the 11 IOOS Regional Associations (RAs) in the US, and its ongoing involvement in the project suggests that there may be untapped opportunities to extend this tool to other IOOS RAs and perhaps expand its coverage to include more of the kinds of data streams described here. If accompanied by establishment of robust connections with public health organizations and academic medical and environmental health institutions, such expansion could effectively create a regionally-based, coastal health observing system. The IOOS program already has well established data and metadata standards and data management protocols, and each RA operates with a governing board that potentially could be expanded to include people with expertise in human health and health data.

### 4.2. Big data considerations

“Big Data” and artificial intelligence (IA) approaches show extraordinary potential for application in environment and health contexts (32, 165). Application of “Big Data” methods in a health observing system context could lead to detection of new causal connections among environmental factors and specific positive and negative health outcomes, discovery of mechanisms of action, and identification of coastal health “hot spots” where conditions favor better or worse health for residents and visitors. However, numerous challenges and impediments remain, not only for Big Data but also for data assimilation across disciplinary divides such as proposed here. Among others, these include: differing scientific cultures and data management processes among disciplines, lack of standardization in data collection and management, disparities in temporal and spatial scales, fragmentation of data sets, shortages of trained personnel, technical resources, and especially funding, data hoarding and reluctance to share, regulatory and proprietary requirements for data privacy, and enforceable commitments to equity in data collection, sharing, and use. In a health context, sharing and assimilating health data while maintaining data security and confidentiality are exceptionally thorny issues. Also, a major gap is “*a realistic estimate of the essential infrastructure, true costs, and other factors needed for sustained collection, storage, analysis, and sharing of big data around the issue of environment and health*” (165, p. 10). Lessons learned from implementation of a coastal health observing system, even at a pilot scale, could help fill this gap by providing specific information about resources necessary to carry out larger-scale efforts to link environmental and health data.

### 4.3. Related approaches

The observing system proposal provides a conceptual model of how environmental and human health data could be connected to provide a more holistic view of health of an individual, community or other group. In this regard, it joins numerous other frameworks that conceptualize ways to connect environmental and human health information, several of which are based on the original DPSIR (Driver-Pressure-State-Impact-Response) model. Among these are the EBM-DPSER (Ecosystem Based Management-Driver-Pressure-State-Ecosystem Service-Response) model (166), the DPSEEA (Driver-Pressure-State-Exposure-Effect Action) and eDPSEEA (ecosystem enriched) models of (167, 168), and the disaster-focused DPSERH (Disaster-Pressure-State-Ecosystem Service-Response-Health) model (169). Such conceptual models, along with others such as the wellbeing indicator framework of (170), can help drive research and policy. The coastal environmental and human health observing system model, while similar to these in many respects, differs significantly in that it includes a substantial amount of individually-derived health data and it focuses on geographic areas and populations that are particularly vulnerable to effects of global environmental change. The proposed observing system also may be useful in the development of health impact assessments (HIAs). HIAs are typically viewed as constructs for public health applications such as evaluating the likely health implications of ongoing or future modifications to the environment, whether purposeful or the result of disasters or accidents, or to changes in health and social policies. While not specifically targeted here, the observing system could provide data in support of HIA development in a variety of situations.

One attempt to harness health and environmental data at a global scale is a proposal for a “Planetary Health Watch (PHW)” system (171). This work is in response to concerns that human activities could exceed one or more of nine planetary boundaries, with resulting abrupt environmental changes and calamitous impacts on human wellbeing (172). Of particular concern are the boundaries associated with climate change, ecosystem integrity, and nitrogen and phosphorus flows. Belesova et al. (171) pointed out the need for integrated monitoring efforts to better understand and mitigate risks associated with increasing pressures on planetary boundaries and called for the creation of a PHW system that would include data from local as well as global scales. If established, coastal health observing systems could provide important local level input for a global PHW system.

While collecting, linking and integrating a broad range of data are essential, development of an operational coastal environmental and health observing system will also require much effort along several other fronts. These include (1) engagement of and active participation by diverse groups of scientists, practitioners, and community members; (2) the breaching of heavily siloed disciplinary and institutional strongholds with extensive cross-piping for bi-directional flows of people, data, and ideas; and (3) the creation of significant financial and professional incentives (e.g., grants, institutional recognition) to attract inter- and trans-disciplinary warriors willing to cross boundaries and build alliances. Students will likely be among the most willing if there is funding to support their education in such endeavors.

### 4.4. Expected benefits of the observing system

Overall, significant benefits from the proposed coastal health observing system are expected to accrue to individual participants, public health, biomedical, and environmental practitioners and researchers, communities, the military, and the public at large. For individuals these may include regular medical check-ups and routine updates on one’s overall health status, discovery and subsequent treatment of unrecognized health problems, information that might be useful for insurance or other purposes, enhanced ability to deal with stress, and increased capacity to control potentially beneficial and harmful exposures for themselves, their families, and communities. Having much more information about potential health-associated effects could help communities and the military design and implement more health promoting and protecting policies, activities, and public spaces and improve overall community resilience to negative effects of global change. Benefits for public health, biomedical, and environmental practitioners and researchers will include much deeper understanding of health benefits and risks associated with living and visiting coastal areas, linking of cause and effect for certain health outcomes, and development of more effective interventions and mitigation methods.

## 5. Recommendations for action

These recommendations are solely those of the author and do not reflect any discussions with or agreement by any other persons or entities.

Recommendations 1 and 2 are for action at a high governmental level and would likely require some time for results to present. Recommendation 3 could be accomplished more quickly if there is interest in the agencies identified. Recommendation 4 is for a grassroots effort to bring attention to the need and to develop support in the US Congress and Federal and State agencies over time.

**Recommendation 1:** The Secretary of the US Department of Health and Human Services (HHS) or other Cabinet level leader designated by the President, could convene directors of HHS agencies (e.g., the NIH and CDC), and request participation of administrators of other Federal agencies with interests in the area including the Department of Defense (DoD), EPA, NOAA, and the National Science Foundation (NSF), plus representatives of State, County, and local public health and environmental agencies to develop and implement a national strategy for integrating available health and environmental condition data and collecting additional data as may be required. Such effort should include standardization of data collection, management and sharing; data quality improvement; engagement of diverse communities and interested collaborators including philanthropic organizations and underserved communities; and funding options. As a first step, the assembled agencies or a subset could commission a consensus study to be conducted via the National Academies of Science, Engineering, and Medicine. Results of the consensus study could be used as guidance for initiating the observing system [Note this recommendation is somewhat similar to Recommendation 3-1 in (82)].

**Recommendation 2**: The European Marine Board (EMB), could address similar issues as those noted above, with a goal to develop a pan-Europe coastal environmental and health observing strategy. The EMB has been a strong supporter of OHH research since at least 2013 (173), and this might be a logical next step. Similar actions could be taken wherever else in the world there may be governmental interest.

**Recommendation 3**: The Administrator of NOAA, working with Directors/Administrators of EPA, NIH, and NSF, could direct senior staff to explore options to incorporate health and health-relevant environmental data into the Integrated Ocean Observing System as suggested here. As first steps, one or more pilot projects could be designed and implemented by interested IOOS Regional Associations.

**Recommendation 4:** Individual scientists, groups of scientists, or scientific organizations could organize community input through development and dissemination of a comprehensive “white paper” or publication that could serve as a vehicle for engaging with governmental funding agencies, legislative bodies, non-governmental organizations, and the private sector. This kind of process can provide significant impetus for initiating program development.

## 6. Conclusion

Notwithstanding the discouraging array of barriers and impediments to integrating environmental and human health data via an observing system, we need to begin somewhere. Coastal areas seem to be a good starting place since they are especially vulnerable to climate change, have high population levels, significant environmental observation resources, particularly for some environmental factors that directly affect human health (e.g., water pollution, HABs, major storms, rip currents), and expose people to both health promoting and health threatening factors. The coastal human health observing system described here would link a variety of coastal environmental observations and health data for individuals, communities, and where possible cohorts. Over time, establishment of a network of such studies focused on residents of coastal areas could provide powerful tools for understanding the health effects of coastal living, good and bad, and lead to better health protections and enhanced wellbeing. While only aspirational at this time, an interconnected system of coastal human health observatories could provide significant benefits to coastal residents, including those in environmental justice and other disadvantaged communities. The basic elements for a coastal health observing system exist but need to be connected, integrated, supported, and implemented. Significant progress could be made in incremental fashion by adding a few health observations to some well-established and environmental observing systems and by augmenting existing health monitoring programs with some environmental observations.

## Data availability statement

Publicly available datasets were analyzed in this study. No new data were collected and no existing data were analyzed. The paper is a thought study based on literature and experience.

## Author contributions

The author confirms being the sole contributor of this work and has approved it for publication.

## Funding

Partial support for this study was provided by the National Institute of Environmental Health Sciences (NIEHS) under award number P01ES028942 to the University of South Carolina and a sub-award to the College of Charleston.

## Conflict of interest

The author declares that the research was conducted in the absence of any commercial or financial relationships that could be construed as a potential conflict of interest.

## Publisher’s note

All claims expressed in this article are solely those of the authors and do not necessarily represent those of their affiliated organizations, or those of the publisher, the editors and the reviewers. Any product that may be evaluated in this article, or claim that may be made by its manufacturer, is not guaranteed or endorsed by the publisher.
